# Precision nutrition in sports science: an opinion on omics-based personalization and athletic outcomes

**DOI:** 10.3389/fnut.2025.1611440

**Published:** 2025-06-06

**Authors:** Mirza Hapsari Sakti Titis Penggalih, Yosef Stefan Sutanto, Nurpudji Astuti Taslim, Rony Abdi Syahputra, Hardinsyah Hardinsyah, Raymond Rubianto Tjandrawinata, Fahrul Nurkolis

**Affiliations:** ^1^Department of Nutrition and Health, Faculty of Medicine, Public Health, and Nursing, Universitas Gadjah Mada, Yogyakarta, Indonesia; ^2^Department of Physical Medicine and Rehabilitation, Prof. R. D. Kandou General Hospital, Sam Ratulangi University, Manado, Indonesia; ^3^Division of Clinical Nutrition, Department of Nutrition, Faculty of Medicine, Hasanuddin University, Makassar, Indonesia; ^4^Department of Pharmacology, Faculty of Pharmacy, Universitas Sumatera Utara, Medan, Indonesia; ^5^Division of Applied Nutrition, Department of Community Nutrition, Faculty of Human Ecology, IPB University, Bogor, Indonesia; ^6^Center for Pharmaceutical and Nutraceutical Research and Policy, Faculty of Biotechnology, Atma Jaya Catholic University of Indonesia, Jakarta, Indonesia; ^7^Master of Basic Medical Science, Faculty of Medicine, Universitas Airlangga, Surabaya, Indonesia; ^8^State Islamic University of Sunan Kalijaga (UIN Sunan Kalijaga), Yogyakarta, Indonesia; ^9^Medical Research Center of Indonesia, Surabaya, East Java, Indonesia

**Keywords:** precision nutrition, omics, sports science, personalized nutrition, athletic performance, recovery, multi-omics integration

## 1 Introduction

Precision nutrition has emerged as a rapidly evolving interdisciplinary field within sports science, presenting an innovative approach to tailoring nutritional strategies for optimizing athletic performance (1–3). By harnessing omics technologies such as nutrigenomics, metabolomics, proteomics, and transcriptomics, precision nutrition delves into the molecular and metabolic nuances of athletes (4). These advancements pave the way for individualized nutritional interventions that consider genetic, biochemical, and environmental variations, moving beyond traditional, generalized dietary recommendations. In a landscape increasingly shaped by data-driven approaches, the application of omics-based precision nutrition has the potential to refine strategies for training adaptation, recovery, injury prevention, and performance enhancement (5).

This review explores the intersection of precision nutrition and sports science, highlighting the role of omics technologies in crafting personalized dietary solutions for athletes. By addressing three key research questions, the review synthesizes and critically evaluates existing literature: how can omics technologies be utilized to optimize personalized nutrition strategies for athletic populations? What evidence supports the effectiveness of such interventions in enhancing performance, recovery, and injury prevention? And what challenges and opportunities are encountered when translating omics-based findings into practical applications within sports settings? Addressing these questions provides a foundation for understanding how molecular-level insights can be applied to achieve measurable benefits for athletes.

The methodology employed in this review includes a comprehensive analysis of existing peer-reviewed literature, spanning studies on genomics, proteomics, metabolomics, and transcriptomics, as well as systematic reviews and clinical trials (6). By integrating findings from diverse studies, the paper adopts a narrative approach to present a cohesive understanding of omics-based precision nutrition. Key analytical methods include critical evaluation, comparative analysis of technological applications, and reflection on methodological advancements and limitations. This approach ensures a balanced discussion that underscores areas of progress while acknowledging remaining challenges in the field.

Current research demonstrates the promising potential of omics technologies to enhance sports nutrition. For example, metabolomics has advanced the understanding of metabolic pathways such as fatty acid oxidation and glycolysis in exercise physiology, while nutrigenomics has identified genetic markers associated with athletic traits such as endurance and injury susceptibility (7, 8). Proteomics has further enabled insights into protein expression changes during recovery and adaptation (9). However, challenges persist in translating these findings into scalable and actionable interventions, with limitations stemming from methodological inconsistencies, ethical considerations, and the complexity of integrating multi-omics data.

The structure of this paper is designed to provide a thorough exploration of the topic. Following this introduction, Chapter 2 focuses on the specific omics technologies employed in sports nutrition, outlining their methodologies, applications, and limitations. Chapter 3 transitions to the practical applications of these technologies, examining their impact on athletic performance, recovery, and injury prevention, while also addressing the challenges of implementation and future research directions. The paper concludes by synthesizing the findings and underscoring the transformative potential of precision nutrition in sports science, while reflecting on the practical, ethical, and methodological hurdles that must be addressed to advance this field.

### 1.1 Literature search strategy

Although this work adopts a narrative review approach, a structured literature search was conducted to ensure comprehensive coverage of relevant studies. Databases including PubMed, Scopus, and Web of Science were searched for peer-reviewed articles published between 2005 and 2024 using keywords such as “precision nutrition,” “sports genomics,” “nutrigenomics in athletes,” “metabolomics exercise,” and “multi-omics sports science.” Articles were selected based on their relevance to the application of omics technologies in sports performance, recovery, or injury prevention. Emphasis was placed on recent advancements, clinical trials, expert opinions, and systematic reviews. Duplicates and studies with low methodological quality or unclear relevance were excluded during the screening process.

## 2 Omics technologies in sports nutrition

**Figure 1:**
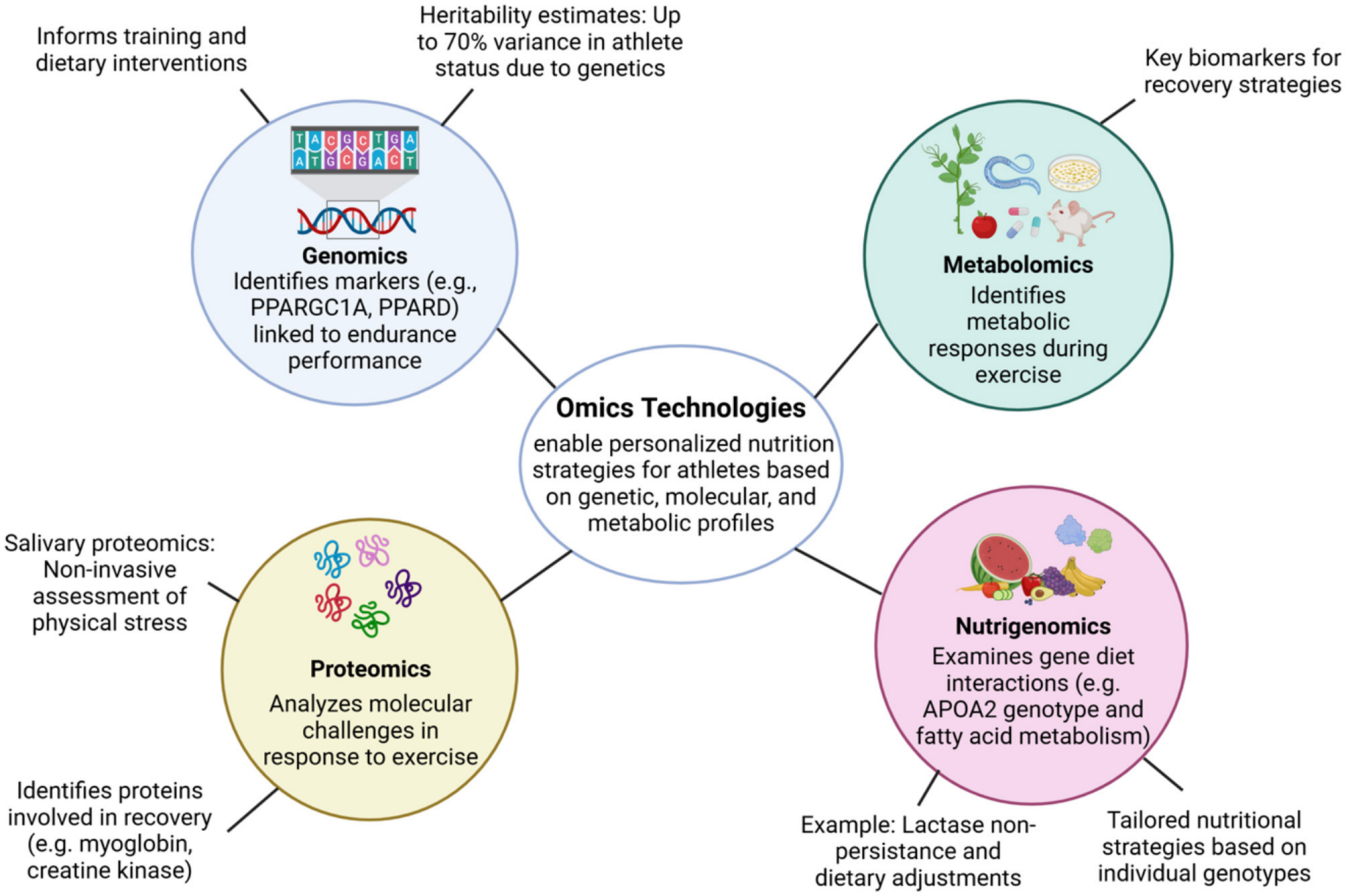
Omics technologies in sports nutrition. Overview of omics technologies and their contributions to precision nutrition in sports. Genomics informs endurance potential and injury risk; nutrigenomics guides dietary adjustments based on gene-diet interactions; proteomics reveals molecular responses and recovery markers; metabolomics identifies real-time metabolic shifts during exercise. The integration of these omics enables the formulation of personalized nutrition strategies tailored to each athlete's unique profile.

Omics technologies have revolutionized sports nutrition by enabling personalized dietary and training strategies based on individual genetic, molecular, and metabolic profiles. By integrating various omics domains—genomics, proteomics, metabolomics, and beyond—researchers can develop precision nutrition interventions tailored to optimize athlete performance and recovery (Figure 1).

### 2.1 Genomics and athletic potential

#### 2.1.1 Genetic markers and performance traits

Advancements in genomics have identified specific genetic markers that are closely linked to athletic performance traits. Genes such as PPARGC1A, which regulates mitochondrial biogenesis, and PPARD, involved in lipid metabolism and muscle fiber composition, are particularly associated with endurance capabilities (10–12). These markers underline the genetic basis that governs traits such as muscle efficiency and energy metabolism. By leveraging these insights, genomic profiling can inform training programs and dietary interventions that align with an athlete's genetic predispositions (13). However, the practical translation of this information into actionable recommendations requires further refinement to increase its accessibility and usability for both sports practitioners and athletes (14, 15).

#### 2.1.2 Heritability and talent identification

The heritability of elite athletic performance, with estimates attributing up to 70% of variance in athlete status to genetic factors, emphasizes the crucial role of genomics in understanding athletic potential (16). This high degree of genetic influence supports the use of genomic screening for early talent identification and strategic athlete development. However, heritability studies often do not account for environmental influences, such as training and nutrition, which interact with genetic predispositions to shape performance outcomes. The complexity of these gene-environment interactions necessitates more integrative research approaches to contextualize heritability findings (17).

#### 2.1.3 GWAS and methodological challenges

Genome-wide association studies (GWAS) have significantly contributed to our understanding of the genetic architecture of athletic traits by identifying hundreds of potential performance-related single nucleotide polymorphisms (SNPs) (18–20). However, these studies often face reproducibility issues due to small sample sizes, ethnic homogeneity, and confounding variables. Initiatives like the Athlome Project Consortium underscore the importance of large and diverse sample sizes to address these challenges. Despite technological advancements, the inability to consistently identify significant genetic variants highlights a limitation of current GWAS methodologies (21–23). This shortcoming points to the need for innovative study designs that go beyond statistical associations to elucidate causal relationships underlying athletic traits (17, 24).

The transition from candidate gene approaches to hypothesis-free GWAS methodologies has marked a significant evolution in sports genomics. By reducing bias and expanding the scope of inquiry, this shift holds promise for uncovering novel genetic factors linked to traits like VO_2_max and injury susceptibility (25, 26). Nonetheless, the field must address challenges such as data interpretation and the functional validation of identified genetic variants to ensure meaningful applications in sports contexts (24).

#### 2.1.4 Ethical considerations in sports genomics

The application of genomics raises important ethical considerations, particularly regarding data misuse, privacy concerns, and potential genetic discrimination (27). Athletes may face risks if sensitive information about their genetic predispositions is improperly disclosed or used to their detriment (28). Establishing clear ethical guidelines and robust policies is imperative for ensuring that genomics is applied responsibly and equitably in sports settings (29).

### 2.2 Nutrigenomics and dietary personalization

#### 2.2.1 Gene-diet interactions and nutrient metabolism

Nutrigenomics, which examines the interaction between genetic variations and dietary needs, has demonstrated the potential for optimizing nutritional strategies based on individual genotypes (30–32). For example, the APOA2 genotype influences fatty acid metabolism, and specific dietary fat intakes can modify its effects, thereby affecting athletic performance (33, 34). By identifying genotypes linked to nutrient processing efficiencies or sensitivities, practitioners can design diets that align with each athlete's metabolic profile. These approaches promise improvements in energy utilization, body composition, and overall performance, although robust validation through long-term, controlled studies remains necessary (35).

#### 2.2.2 Lactase non-persistence and gut microbiota

Another example of the value of nutrigenomics is seen in individuals with genetic lactase non-persistence, who exhibit altered gut microbiota and metabolite concentrations when consuming high amounts of milk (36, 37). Tailoring dietary recommendations to avoid or limit lactose in such individuals not only mitigates discomfort but also enhances metabolic health and nutrient absorption (38). This precision approach exemplifies how genetic information can be used to refine nutritional guidance in ways that support both health and athletic performance, particularly through the modulation of gut microbiome function. Yet, further investigation is needed to establish standardized protocols for such applications (35).

#### 2.2.3 SNPs and personalized dietary planning

The identification of over 50,000 single nucleotide polymorphisms (SNPs) influencing cellular functions provides a vast dataset for crafting athlete-specific diets. These diets can address genetic predispositions impacting recovery, metabolism, and training adaptations. However, making this information actionable requires the development of user-friendly tools and platforms that can translate complex genetic data into practical recommendations for coaches and nutritionists (29, 30).

#### 2.2.4 Nutrient deficiencies and supplementation

Nutrigenomics has also shown promise in optimizing macronutrient and micronutrient strategies for athletes. For instance, by identifying genetic susceptibilities to nutrient deficiencies, such as specific vitamin or mineral needs, tailored supplementation can be offered, potentially improving performance and recovery. Athletes with polymorphisms affecting vitamin D, iron, or folate metabolism may require personalized intake strategies to maintain optimal physiological status (38, 39). This proactive approach can enhance immune function, reduce fatigue, and expedite recovery, offering competitive advantages. Nonetheless, the integration of these insights into sports nutrition programs is currently hindered by high testing costs and the lack of standardized implementation protocols across athletic organizations (40).

### 2.3 Proteomics and exercise adaptation

#### 2.3.1 Molecular changes during exercise

Proteomics analyzes molecular-level changes in response to exercise, shedding light on the physiological processes involved in adaptation and recovery (41, 42). The upregulation of proteins such as myoglobin and creatine kinase highlights their roles in muscle repair and immune response following intense activity (43). These findings provide a foundation for targeted nutritional and recovery interventions but require further research to elucidate how dietary factors can specifically modulate these protein responses (44).

#### 2.3.2 Salivary proteomics and recovery assessment

Salivary proteomic profiling has revealed a substantial increase in total salivary proteins after prolonged exertion, presenting a non-invasive method for assessing physical stress and recovery states (45, 46). While promising, the applicability of salivary biomarkers in diverse athletic contexts needs further validation to establish their reliability and utility across different sporting disciplines (47). Exercise-induced modifications to protein structures, such as lysine acetylation, play crucial roles in regulating mitochondrial function and energy production, both essential for endurance performance (48). Understanding these molecular mechanisms can inform training programs aimed at enhancing mitochondrial efficiency; however, translating these insights into actionable recommendations for athletes is still in the early stages (49).

Proteomic data has demonstrated how environmental factors, such as altitude, influence protein expression, enabling tailored strategies for athletes exposed to variable conditions (50, 51). For instance, specific dietary or training adaptations can be designed to mitigate performance declines at high altitudes. Integrating such insights into practice necessitates more comprehensive research on the interaction between environmental stressors and proteomic responses (52). Nutrition-centered proteomic studies have linked specific dietary interventions to enhanced recovery pathways in endurance athletes. These findings underscore the potential of proteomics to optimize performance; however, the development of cost-effective and scalable methods for analyzing proteomic data is critical for broader application in sports nutrition (7).

#### 2.3.3 Post-translational modifications and mitochondrial efficiency

Metabolomics has provided critical insights into metabolic responses during exercise, identifying key biomarkers like lactate and pyruvate that signal energy depletion (53, 54). Nutritional strategies based on these biomarkers can enhance recovery by replenishing energy reserves more efficiently. However, standardization of analytical methods is necessary to ensure consistency across studies and practical applications (55). Post-exercise metabolomic profiling has identified changes in tricarboxylic acid (TCA) cycle intermediates, ketone bodies, and lipid metabolites, which reflect recovery efficiency (56, 57). These findings can inform targeted dietary interventions, but challenges like small sample sizes and variability in metabolic responses limit the generalizability of these results (58).

The discovery of miR-532-5p as a biomarker for training adaptations demonstrates the potential of metabolomics to predict and monitor individual responses to exercise. While promising, the application of such biomarkers requires further validation to confirm their reliability and reproducibility in diverse athletic populations (59). Changes in metabolic pathways, such as shifts toward fatty acid metabolism and reduced glycolysis, are indicative of long-term adaptations to exercise that improve energy efficiency (60–63). Precision nutrition strategies can leverage these insights to design training and dietary programs that maximize these benefits, though continued research is needed to optimize their integration (64). Lipidomics, a subset of metabolomics, has highlighted differential tissue-specific lipid metabolism responses to exercise, emphasizing the importance of tailoring endurance strategies based on individual metabolic profiles (56, 65). However, the limited scalability of lipidomics due to cost and analytical requirements restricts its broader adoption (66).

#### 2.3.4 Relevance of omics technologies

Multi-omics integration offers a holistic approach by combining datasets from genomics, proteomics, and metabolomics, among other fields, to derive a comprehensive understanding of athletic physiology (67–71). This approach supports the identification of precise, personalized interventions aimed at improving performance and health outcomes. The adoption of integrative platforms and emerging tools, such as OmicsAnalyst and artificial intelligence, facilitates the analysis of complex datasets, enabling improved precision in tailoring nutrition and training strategies (72, 73). Yet, challenges such as ethical concerns, logistical hurdles, and data integration complexities persist, limiting the broader implementation of multi-omics approaches in real-world settings (74, 75).

Further highlighting the relevance of multi-omics, its role in individualized athlete monitoring is amplified by advancements in wearable technology and biosensors, which allow the real-time collection of metabolic and physiological data. Such technological innovations bridge the gap between laboratory-based analyses and everyday training environments, offering unprecedented opportunities for personalized interventions. However, the high costs and need for multidisciplinary cooperation to interpret these data remain substantial obstacles (76, 77).

The scalability of omics-based interventions remains a pressing issue, particularly given the limited accessibility of sophisticated technologies for amateur or under-resourced athletic groups. However, the potential for technological advancements, such as low-cost real-time monitoring systems, may gradually resolve these limitations, making precision nutrition more widely available. Continued efforts to improve the usability and affordability of multi-omics platforms will be vital in democratizing access to these tools, thereby broadening their impact across diverse athletic populations (78–80).

Omics technologies offer immense potential for advancing sports nutrition, but addressing challenges related to cost, data integration, and ethical considerations is essential for their widespread application. Further research aimed at overcoming these limitations will be critical to maximizing the utility of omics-based precision nutrition in sports science.

## 3 Applications in athletic performance and recovery

Metabolomic profiling during aerobic activity has identified significant changes in metabolite levels, including oxaloacetate and tyrosine, which are critical for energy production and mitochondrial function (44, 81–83). These metabolic shifts are particularly pronounced under hypoxic conditions, such as those encountered at high altitudes. Oxaloacetate is a crucial intermediate in the tricarboxylic acid (TCA) cycle, playing a vital role in energy metabolism, while tyrosine contributes to neurotransmitter synthesis and overall metabolic regulation during physical exertion (84, 85). Tailored nutritional strategies that replenish these depleted intermediates can be employed to optimize recovery and support sustained performance during high-intensity activities. However, standardized methodologies for assessing these metabolite changes in diverse athletic populations remain lacking, limiting the broader applicability of such interventions. Future studies should prioritize larger sample sizes and account for individual variability in response to metabolic stress to refine these approaches (44).

### 3.1 Nutritional strategies for high-altitude performance

**Figure 2:**
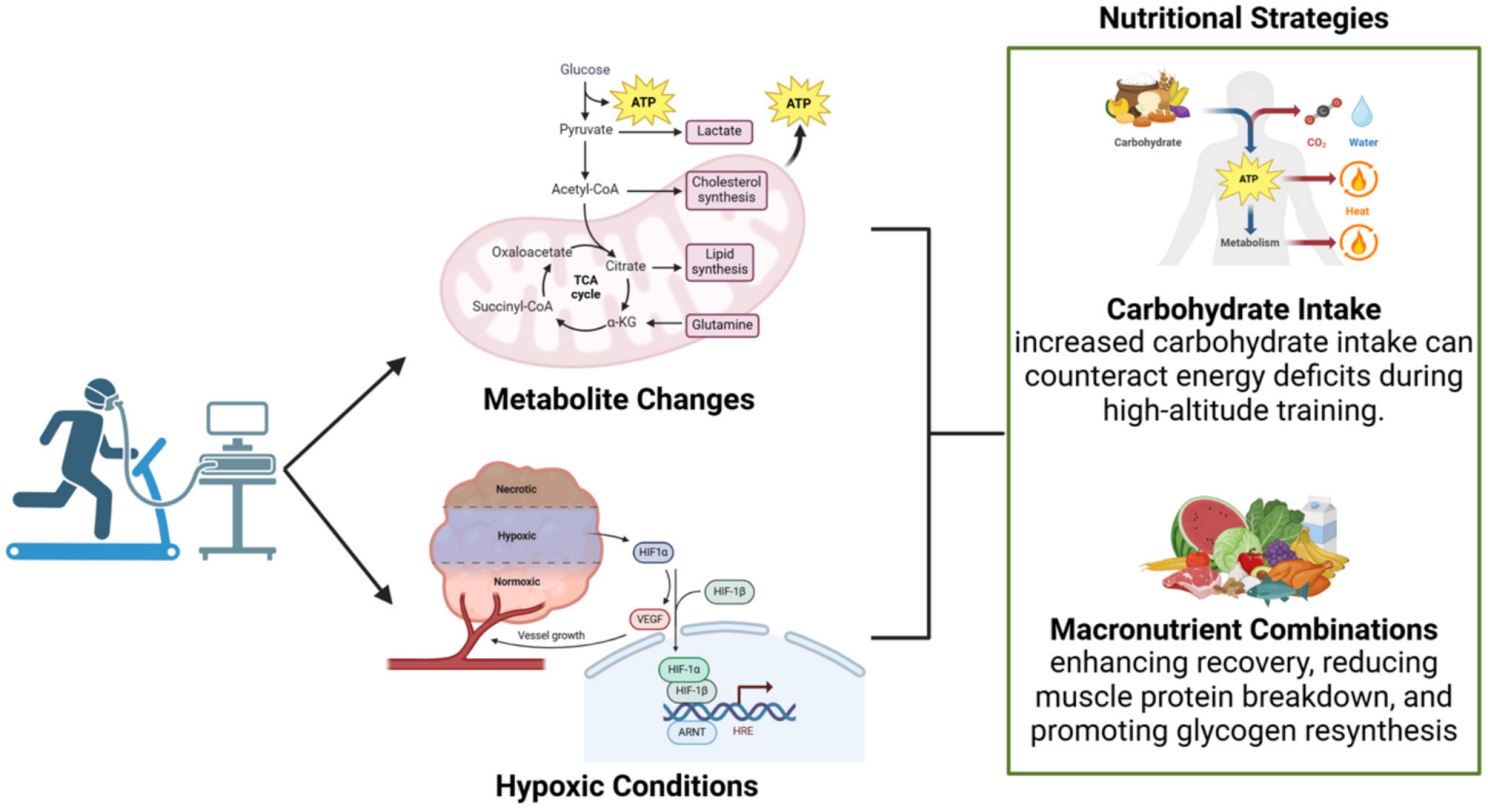
Metabolic changes and nutritional strategies during aerobic activity. Aerobic exercise under hypoxic conditions induces metabolite changes, such as elevated lactate and shifts in the TCA cycle, which influence energy production and recovery. These alterations highlight the importance of personalized nutritional strategies, such as increased carbohydrate intake to mitigate energy deficits at high altitudes and macronutrient combinations to enhance muscle recovery and glycogen resynthesis.

Hypoxic conditions further exacerbate systemic recovery challenges, as evidenced by metabolomic profiles that provide specific insights into altitude-specific nutritional requirements (Figure 2). These conditions necessitate tailored recovery protocols, with a particular focus on dietary interventions aimed at mitigating the physiological stresses imposed by reduced oxygen availability (86, 87). For instance, high-altitude training could benefit from increased carbohydrate intake to counteract energy deficits and reduce reliance on protein catabolism for energy. Despite these promising applications, the complexities of individual metabolic responses to hypoxia underscore the need for personalized approaches and more precise analytical tools to support evidence-based recommendations (44).

### 3.2 Biosensors and machine learning for real-time nutritional feedback

Advancements in wearable technology and mobile sensors now allow for real-time analysis of biomarkers such as sweat metabolites, offering immediate feedback on hydration levels and substrate utilization. These innovations enable athletes and their support teams to make on-the-spot adjustments to hydration and dietary intake, improving training and competition outcomes. The integration of biochemical data with digital platforms also bridges critical gaps in traditional nutritional monitoring methods (88–92). However, the implementation of wearable technology faces challenges, including variability in data accuracy and the complexity of interpreting results in real-world settings. Future research should explore ways to enhance the reliability of these devices while simultaneously simplifying their use for athletes and coaches (22).

The amalgamation of wearable technologies with machine learning algorithms further enhances decision-making by identifying patterns in metabolic responses unique to each athlete. These data-driven insights facilitate the creation of highly specific nutrition plans that align with individual physiological needs (93, 94). While the potential for machine learning to revolutionize personalized nutrition is substantial, the success of these tools depends on the availability of robust datasets and multidisciplinary collaboration to interpret complex interactions between genetics, environment, and training. Additionally, ethical considerations regarding data security and athlete privacy must be addressed before these technologies can be widely adopted in high-performance settings (22).

### 3.3 Post-exercise recovery and muscle repair

Post-exercise nutritional interventions involving macronutrient combinations, particularly proteins and carbohydrates, have demonstrated the ability to enhance recovery by simultaneously reducing muscle protein breakdown and promoting glycogen resynthesis (95–97). Low-carbohydrate, protein-rich beverages have been shown to modulate catabolic and anabolic markers effectively, with reductions in 3-methylhistidine levels indicating decreased muscle protein degradation and increases in pseudouridine signaling improved cellular recovery (98, 99). While promising, these findings reveal the complexity of optimizing recovery strategies for varying athletic demands and fitness levels. Athletes with lower fitness levels, for example, may benefit disproportionately from such tailored macronutrient interventions, as their recovery states are often more metabolically compromised. Nonetheless, the practical implementation of these dietary strategies requires further investigation into individual variability and long-term efficacy (100).

Proteomic analyses have revealed that post-exercise recovery is associated with the upregulation of specific proteins, including myoglobin and creatine kinase, which play central roles in muscle repair and adaptation. Myoglobin facilitates oxygen transport within muscle cells, while creatine kinase is pivotal in energy homeostasis and cellular repair mechanisms (101, 102). Monitoring these proteins provides valuable insights into how athletes respond to physical stress, offering opportunities to design more targeted recovery protocols. Expanding proteomic applications to include the analysis of immune response proteins could further elucidate how inflammatory processes are managed post-exercise. While these advances hold significant promise, their widespread application is hindered by the high costs and technical expertise required to implement proteomic testing on a larger scale (44, 103).

### 3.4 Metabolic demands of high-intensity exercise in heat

High-intensity exercise conducted in hot environmental conditions induces significant metabolic changes, such as elevated lactate and glucose levels, reflecting increased reliance on anaerobic pathways for energy production (104–106). These findings stress the importance of implementing hydration strategies that address both fluid loss and the heightened metabolic demands associated with heat stress. Specific carbohydrate supplementation regimens that align with environmental factors can mitigate fatigue and support energy efficiency during prolonged activity in such conditions. Nevertheless, further research is needed to establish comprehensive nutritional guidelines that address the interaction between environmental stressors and individual metabolic responses, ensuring these strategies are adaptable across various athletic populations (107).

### 3.5 Enhanced carbohydrate oxidation through mixed supplementation

Research indicates that combining glucose with other carbohydrates, such as fructose, enhances carbohydrate oxidation rates beyond 1 g/min, significantly improving endurance performance during extended physical activity (108). This metabolic enhancement is particularly relevant for athletes engaged in long-duration or high-intensity events. Genetic and metabolic variability among individuals further emphasizes the need for personalized carbohydrate supplementation plans. Tools such as nutrigenomics can identify single nucleotide polymorphisms (SNPs) that influence metabolic pathways, providing a basis for designing individualized macronutrient strategies. However, the complexity and cost of implementing these genomic insights at scale remain substantial barriers to their widespread adoption (109).

### 3.6 Multi-omics integration for comprehensive nutritional insight

Multi-omics approaches, which integrate genomics, proteomics, and metabolomics, allow for comprehensive tracking of an athlete's physiological responses, thereby enhancing the precision of dietary recommendations (110). This holistic perspective supports the design of interventions that not only address immediate performance needs but also facilitate long-term adaptations to training and competition demands. Tools like OmicsAnalyst have significantly advanced the field by enabling the integration of multi-modal datasets, yet their utility is limited by logistical challenges and the expertise required for data interpretation. Future efforts should focus on making these platforms more accessible and user-friendly while addressing ethical and privacy concerns associated with multi-omics data usage (40).

#### 3.6.1 Gender-specific precision nutrition in female athletes

The application of precision nutrition extends to addressing specific challenges faced by athletes, such as menstrual dysfunction in female athletes. Tailored macronutrient modulation and supplementation strategies have been shown to optimize hormonal health and overall performance in this context (111–115). These interventions are particularly valuable for reducing the risk of stress-induced illnesses and supporting recovery from intensive training loads. However, gender-sensitive research in sports nutrition remains underrepresented, highlighting the need for more diverse and inclusive studies to refine these strategies further (116).

#### 3.6.2 Long-term exercise adaptations and systemic health benefits

Long-term exercise has been shown to induce metabolic adaptations, such as improved beta-oxidation of fatty acids and reduced glycolysis reliance, enhancing energy efficiency. These shifts not only improve immediate athletic performance but also contribute to systemic benefits, such as reduced inflammation and better cardiovascular health. Insights into these adaptations can guide more effective training and nutritional strategies tailored to individual metabolic profiles. Moreover, research indicates that the benefits of exercise extend beyond metabolic efficiency, influencing neurological health and reducing the risk of neurodegenerative diseases. For instance, sustained physical activity has been shown to enhance brain-derived neurotrophic factor expression, which plays a protective role in conditions such as Alzheimer's disease. These findings, derived from multi-omics studies, demonstrate the far-reaching physiological advantages of long-term exercise and further emphasize the importance of holistic approaches to athlete training plans (117, 118).

#### 3.6.3 Urinary metabolomics as non-invasive recovery biomarkers

Urinary metabolome changes occurring after strenuous exercise provide valuable indicators of recovery states and potential pathways for intervention. For example, reduced concentrations of specific metabolites highlight the physiological stress imposed by intensive activity, offering markers for targeted recovery strategies (56). Leveraging these biomarkers can enhance the precision of post-competition nutritional protocols, allowing for faster and more effective recovery. However, the transient nature of these metabolic shifts raises questions about their reliability as long-term indicators, necessitating further exploration of their clinical utility (119).

#### 3.6.4 Genetic markers for injury risk and endurance potential

Genetic markers associated with endurance traits and injury susceptibility offer critical insights for developing proactive training and nutrition plans. These markers facilitate the customization of workloads and recovery schedules to align with an athlete's genetic predispositions, thereby reducing the risk of overtraining and related injuries (120, 121). Such targeted strategies not only enhance training outcomes but also safeguard long-term physical health. Despite this potential, challenges in translating genetic findings into actionable recommendations persist, underscoring the need for ongoing research to refine their practical applications (7).

**Figure 3:**
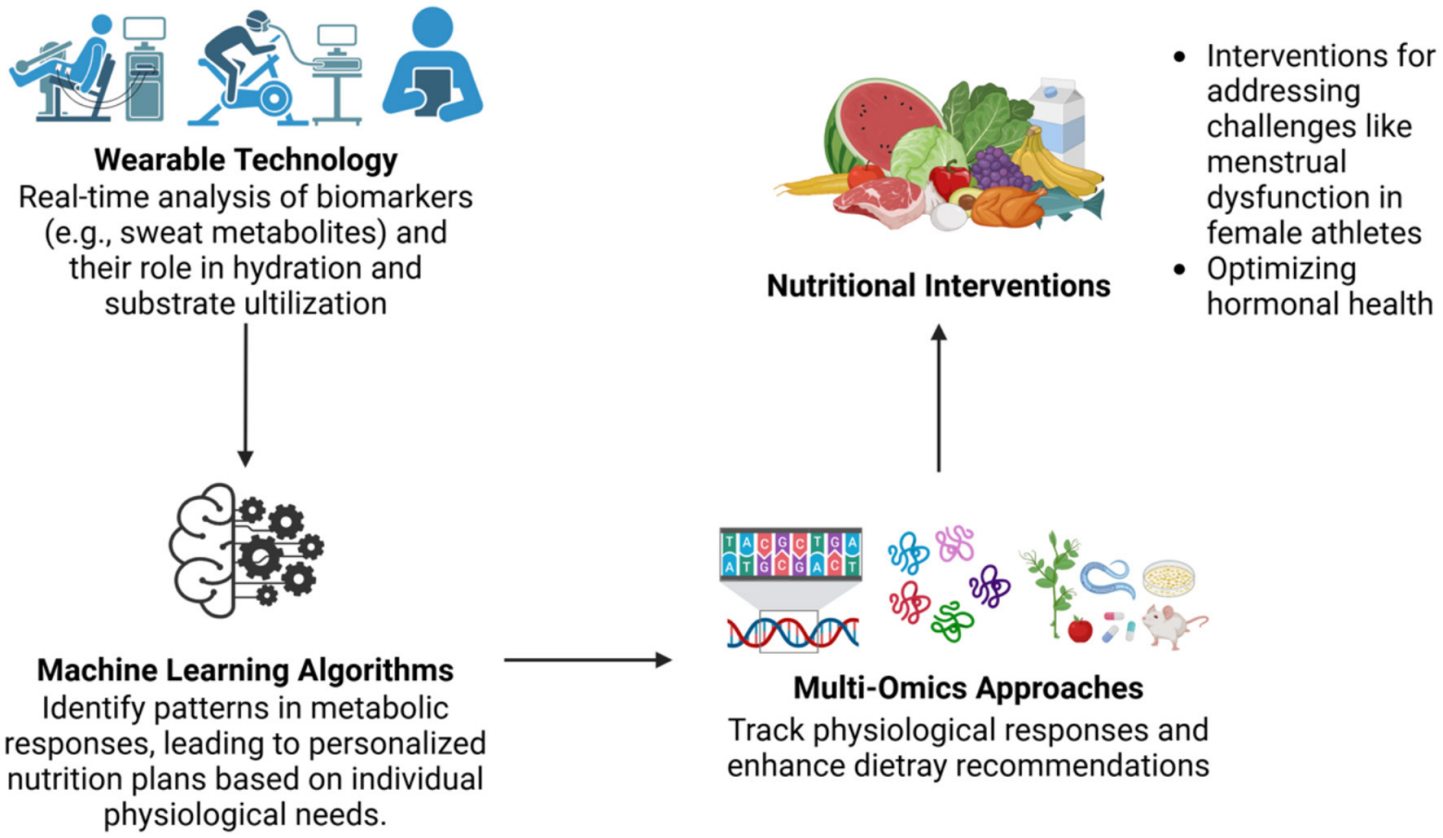
Integration of wearable technology and multi-omics approaches in personalized nutrition. Integration of wearable technologies with machine learning and multi-omics platforms facilitates real-time biomarker monitoring and the development of individualized nutritional interventions. These tools enable precise tracking of physiological responses and support targeted dietary plans, especially in addressing specific issues such as hormonal health and menstrual dysfunction in female athletes.

In conclusion, applying omics technologies to athletic performance and recovery offers significant potential for advancing personalized nutrition strategies (Figure 3). By addressing existing challenges, such as cost, scalability, and data integration, future research can unlock new opportunities to enhance both immediate and long-term athletic outcomes.

## 4 Discussion

The integration of omics technologies into sports nutrition represents a transformative advancement, offering a granular understanding of individual responses to training, recovery, and dietary interventions. Advances across genomics, transcriptomics, proteomics, metabolomics, and microbiomics have enabled the identification of key molecular markers linked to nutrient metabolism, inflammation, muscle adaptation, and fatigue. These technologies have deepened our understanding of how individual genetic variations influence macronutrient utilization, supplement efficacy, and injury susceptibility. For instance, genomics can inform personalized carbohydrate intake strategies, while metabolomics provides real-time snapshots of energy expenditure and recovery status (7). Additionally, gut microbiome profiling offers novel insights into immune modulation and nutrient absorption (122). When combined, these omics approaches form a systems biology perspective that allows for highly individualized nutritional recommendations tailored to the physiological and metabolic demands of athletes. However, despite these scientific advances and pilot applications in elite sports settings, practical translation into routine use remains limited due to issues of standardization, cost, and the need for multidisciplinary expertise. Thus, while the foundational science has made substantial progress in mapping molecular pathways and identifying performance-related biomarkers, we argue that the next phase of development must shift toward pragmatic, scalable, and ethically sound applications that can be implemented across a broader spectrum of athletic populations.

Based on our assessment, we believe that the most urgent future direction is the standardization and validation of multi-omics protocols tailored for athletic populations. This includes harmonizing data collection methods across genomics, proteomics, and metabolomics to allow meaningful comparisons and real-time interpretations. Despite the growing popularity of multi-omics approaches, a lack of standardized bioinformatics pipelines and inconsistencies in sampling procedures limit their reproducibility and translational impact (123). We propose that developing open-access databases and AI-enhanced interpretation frameworks—customized for the athletic context—should become a priority for the research community.

Real-world implementation will depend heavily on cost-effective solutions and interdisciplinary collaboration. Although the current literature demonstrates promising correlations between molecular profiles and performance metrics, translating these insights into actionable strategies remains restricted to elite or well-funded institutions. Wearable biosensors and mobile metabolite-tracking technologies offer an encouraging route to democratize access, but require rigorous validation. We recommend future research prioritize affordability and simplicity of user interfaces, ensuring that omics-driven interventions become accessible to a broader range of athletes, including those in amateur and resource-limited contexts.

We also emphasize that a critical bottleneck lies in the ethical and legal landscape surrounding omics applications. While genomics and other omics data hold immense predictive value, there is an acute risk of misuse, especially in competitive sports environments where pressure to outperform is high. We propose that future studies be accompanied by concurrent development of governance frameworks that address data ownership, informed consent, genetic discrimination, and psychological impacts of predictive findings. Without these, the integration of omics technologies could exacerbate inequities or introduce new forms of athlete exploitation.

Furthermore, we contend that the field needs a more nuanced understanding of gene-environment-nutrition interactions. We believe that focusing exclusively on single molecular domains may obscure synergistic effects that only become apparent through longitudinal, integrative designs. For example, identifying how specific genotypes respond to macronutrient timing under varied environmental stressors—such as altitude or heat—could redefine how training programs are individualized. Such findings will not emerge from siloed research but from multi-center trials incorporating behavioral, physiological, and omics data.

Lastly, we assert that the educational and professional development of practitioners must evolve alongside technological advancements. Nutritionists, trainers, and coaches require upskilling to interpret and implement omics findings responsibly. We propose that curricula in sports science and nutrition incorporate modules on omics literacy, data ethics, and applied systems biology. Only by equipping practitioners with adequate tools can we ensure that precision nutrition transitions from concept to routine practice.

In conclusion, omics-based precision nutrition holds enormous promise but is still in a formative stage of development. We propose that future research prioritize integration, accessibility, ethical rigor, and practitioner training to ensure the field progresses in a sustainable and equitable manner. This vision necessitates not only scientific innovation but also coordinated efforts across disciplines, institutions, and stakeholder communities. Only through such a comprehensive approach can we unlock the full potential of precision nutrition to enhance both performance and well being in diverse athletic populations.

## 5 Key summary

The overarching aim of this work was to investigate how omics technologies—including genomics, nutrigenomics, proteomics, and metabolomics—can enhance precision nutrition to optimize athletic performance, recovery, and injury prevention. Through a synthesis of current research, the study demonstrates how the integration of these molecular approaches facilitates personalized dietary and training interventions tailored to each athlete's unique biological profile. Omics technologies are shown to transform traditional sports nutrition by shifting from generalized recommendations to highly individualized strategies, thereby improving both short-term performance and long-term health outcomes. Genomic analyses have revealed key markers such as *PPARGC1A* and *PPARD* linked to endurance, though challenges such as limited reproducibility in elite populations persist. Nutrigenomics has underscored the influence of genetic predispositions—e.g., *APOA2* variants and lactase non-persistence—on dietary responses, while proteomics and metabolomics have respectively illuminated the roles of exercise-responsive proteins (e.g., creatine kinase, myoglobin) and metabolic biomarkers (e.g., lactate, pyruvate) in recovery and energy balance. Integrating these domains through a multi-omics framework offers a holistic understanding of athletic physiology by capturing the dynamic interplay between genes, proteins, metabolites, and environmental factors. Moreover, this research situates omics within broader efforts in precision medicine, with initiatives like the Athlome Consortium highlighting the value of collaborative biomarker validation. Nonetheless, significant barriers remain, including high implementation costs, complex technological requirements, the need for multidisciplinary data interpretation, and ethical concerns around genetic testing and data privacy. The review also notes that limitations in current literature—such as methodological variability and small sample sizes—hinder generalizability, while the inaccessibility of omics tools to non-elite athletes and the absence of standardized protocols for multi-omics integration constrain practical adoption. Addressing these challenges will be critical to democratizing omics-driven sports nutrition and ensuring its equitable, responsible, and impactful implementation.

**Figure 4:**
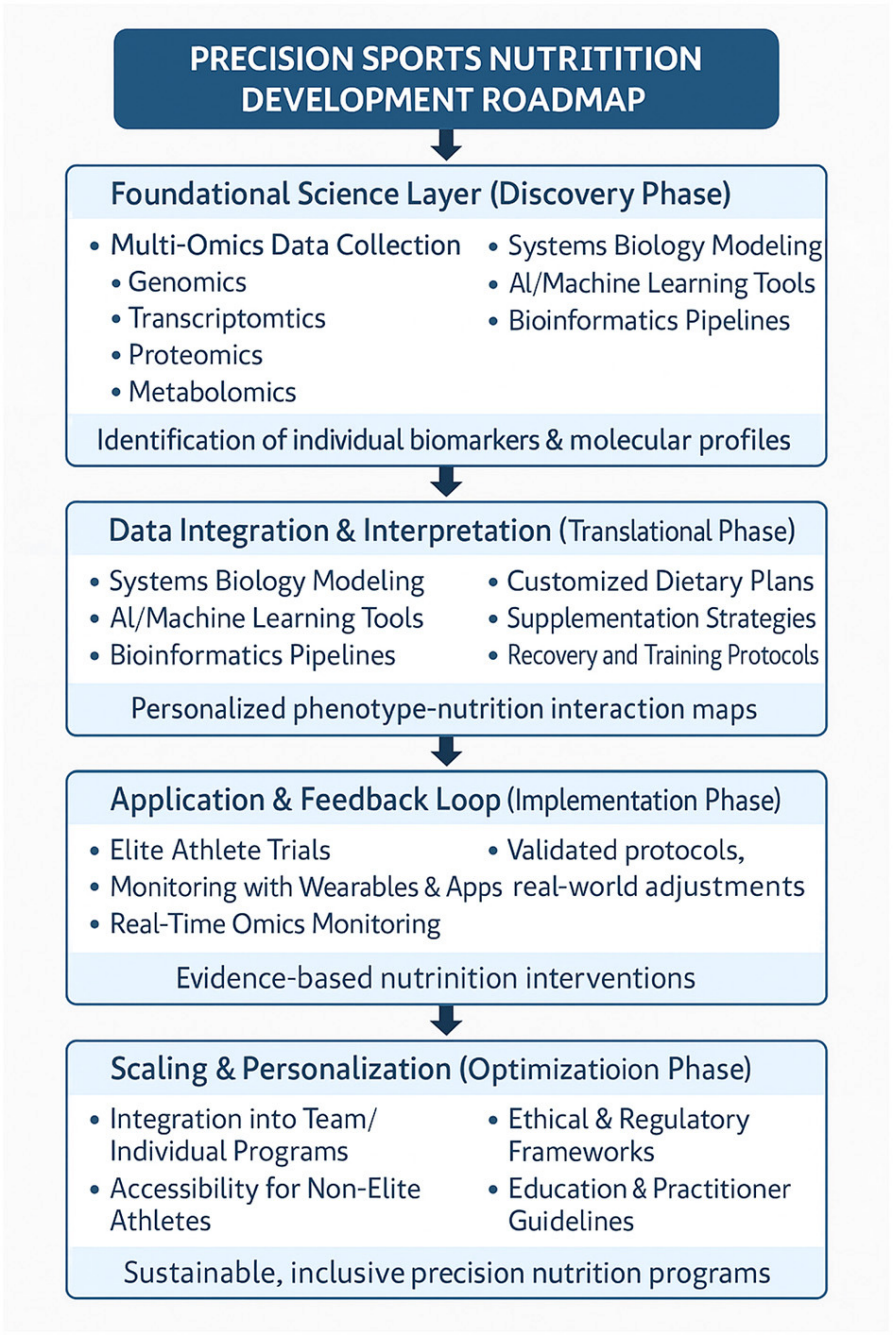
Proposed precision nutrition development roadmap.

Future research must prioritize the refinement of omics technologies to overcome these barriers and expand their applicability (Figure 4). Key areas for exploration include standardizing methodologies for multi-omics integration, developing real-time monitoring systems enabled by wearable biosensors, and enhancing the affordability and usability of omics platforms (Figure 4). Interdisciplinary collaboration among sports scientists, nutritionists, molecular biologists, and data specialists will be critical in transforming omics research into practical solutions. Additionally, greater emphasis on inclusivity and diversity in research is essential to ensure that omics-based strategies are applicable across all athletic populations, including underrepresented groups such as female athletes and those from diverse genetic backgrounds.

Through this work, the immense potential of omics technologies to revolutionize sports nutrition has been highlighted. These approaches not only address immediate performance and recovery needs but also contribute to a more comprehensive understanding of human biology, offering systemic benefits that extend beyond the athletic domain. Writing this review has reinforced the importance of integrating cutting-edge molecular research with practical sports applications, inspiring a commitment to pursue advancements that enhance human potential and well being. By addressing current challenges and fostering interdisciplinary collaboration, omics-based precision nutrition stands poised to set new standards in sports science and personalized health care. This work contributes to that vision by bridging theoretical knowledge with practical implications, establishing a foundation for future research aimed at unlocking the full potential of these transformative technologies.

## References

[1] Nieman DC. Multiomics approach to precision sports nutrition: limits, challenges, and possibilities. Front Nutr. (2021) 8:796360. 10.3389/fnut.2021.796360 34970584 PMC8712338

[2] Exel J Dabnichki P. Precision sports science: what is next for data analytics for athlete performance and well-being optimization? Appl Sci. (2024) 14:3361. 10.3390/app14083361

[3] American Dietetic Association, Dietitians of Canada, American College of Sports Medicine, Rodriguez NR Di Marco NM Langley S. American college of sports medicine position stand. Nutrition and athletic performance. Med Sci Sports Exerc. (2009) 41:709–31. 10.1249/MSS.0b013e31890eb86 19225360

[4] Singar S Nagpal R Arjmandi BH Akhavan NS. Personalized nutrition: tailoring dietary recommendations through genetic insights. Nutrients. (2024) 16: 2673. 10.3390/nu16162673 39203810 PMC11357412

[5] Sanches PHG de Melo NC Porcari AM de Carvalho LM. Integrating molecular perspectives: strategies for comprehensive multi-omics integrative data analysis and machine learning applications in transcriptomics, proteomics, and metabolomics. Biology. (2024) 13:848. 10.3390/biology13110848 39596803 PMC11592251

[6] Rashid A Al-Obeidat F Kanthimathinathan HK Benakatti G Hafez W Ramaiah R. Advancing sepsis clinical research: harnessing transcriptomics for an omics-based strategy - a comprehensive scoping review. Inform Med Unlocked. (2024) 44:101419. 10.1016/j.imu.2023.101419

[7] Bedrač L Deutsch L Terzić S Cervek M Šelb J Ašič U. Towards precision sports nutrition for endurance athletes: a scoping review of application of omics and wearables technologies. Nutrients. (2024) 16:3943. 10.3390/nu16223943 39599728 PMC11597302

[8] Al-Khelaifi FA. In: Elrayess A Abraham D Hingorani A, editors. Genetics and metabolomics of elite athletes: genome-wide association study and metabolomics profiling of elite athletes [Doctoral]. UCL (University College London) (2020). Available online at: https://discovery.ucl.ac.uk/id/eprint/10104070/1/Al-Khelaifi_10104070_Thesis_sig-removed.pdf (accessed May 15, 2025).

[9] Voelckel C Gruenheit N Lockhart P. Evolutionary transcriptomics and proteomics: insight into plant adaptation. Trends Plant Sci. (2017) 22:462–71. 10.1016/j.tplants.2017.03.001 28365131

[10] Bottura RM Dentillo DB. Genomics may be the key to understanding endurance training pillars. Genes. (2025) 16:338. 10.3390/genes16030338 40149489 PMC11942075

[11] Jin H Hwang IW Kim K Cho HI Park TH Shin YA. Is there a relationship between PPARD T294C/PPARGC1A Gly482Ser variations and physical endurance performance in the Korean population? Genes Genom. (2016) 38:389–95. 10.1007/s13258-015-0380-4

[12] Eynon N Meckel Y Alves A Yamin C Michael S Goldhammer E. Is there an interaction between PPARD T294C and PPARGC1A Gly482Ser polymorphisms and human endurance performance? Exp Physiol. (2009) 94:1147–52. 10.1113/expphysiol.2009.049668 19666693

[13] Stefan N Thamer C Staiger H Machicao F Machann J Schick F. Genetic variations in PPARD and PPARGC1A determine mitochondrial function and change in aerobic physical fitness and insulin sensitivity during lifestyle intervention. J Clin Endocrinol Metab. (2007) 92:1827–33. 10.1210/jc.2006-1785 17327385

[14] Ginevičiene V Utkus A Pranckevičiene E Semenova EA Hall ECR Ahmetov II. Perspectives in sports genomics. Biomedicines. (2022) 10:298. 10.3390/biomedicines10020298 35203507 PMC8869752

[15] Spanakis M Fragkiadaki P Renieri E Vakonaki E Fragkiadoulaki I Alegakis A. Advancing athletic assessment by integrating conventional methods with cutting-edge biomedical technologies for comprehensive performance, wellness, and longevity insights. Front Sports Act Living. (2023) 5:1327792. 10.3389/fspor.2023.1327792 38260814 PMC10801261

[16] Semenova EA Hall ECR Ahmetov II. Genes and athletic performance: the 2023 update. Genes. (2023) 14:1235. 10.3390/genes14061235 37372415 PMC10298527

[17] Konopka MJ Sperlich B Rietjens G Zeegers MP. Genetics and athletic performance: a systematic SWOT analysis of non-systematic reviews. Front Genet. (2023) 14:1232987. 10.3389/fgene.2023.1232987 37621703 PMC10445150

[18] Marigorta UM Rodríguez JA Gibson G Navarro A. Replicability and prediction: lessons and challenges from GWAS. Trends Genet. (2018) 34:504–17. 10.1016/j.tig.2018.03.005 29716745 PMC6003860

[19] Visscher PM Wray NR Zhang Q Sklar P McCarthy MI Brown MA. 10 years of GWAS discovery: biology, function, and translation. Am J Hum Genet. (2017) 101:5–22. 10.1016/j.ajhg.2017.06.005 28686856 PMC5501872

[20] Kim J Oh S Min H Kim Y Park T. Practical issues in genome-wide association studies for physical activity. Ann N Y Acad Sci. (2011) 1229:38–44. 10.1111/j.1749-6632.2011.06102.x 21793837

[21] Uffelmann E Huang QQ Munung NS de Vries J Okada Y Martin AR. Genome-wide association studies. Nat Rev Methods Primers. (2021) 1:59. 10.1038/s43586-021-00056-9

[22] Sempionatto JR Montiel VRV Vargas E Teymourian H Wang J. Wearable and mobile sensors for personalized nutrition. ACS Sens. (2021) 6:1745–60. 10.1021/acssensors.1c00553 34008960

[23] Abdellaoui A Yengo L Verweij KJH Visscher PM. 15 years of GWAS discovery: realizing the promise. Am J Hum Genet. (2023) 110:179–94. 10.1016/j.ajhg.2022.12.011 36634672 PMC9943775

[24] Pitsiladis YP Tanaka M Eynon N Bouchard C North KN Williams AG. Athlome Project Consortium: a concerted effort to discover genomic and other omic markers of athletic performance. Physiol Genomics. (2016) 48:183–90. 10.1152/physiolgenomics.00105.2015 26715623 PMC4773890

[25] Alqudah AM Sallam A Stephen Baenziger P Börner A. GWAS: fast-forwarding gene identification and characterization in temperate cereals: lessons from Barley - a review. J Adv Res. (2020) 22:119–35. 10.1016/j.jare.2019.10.013 31956447 PMC6961222

[26] Koenen KC Duncan LE Liberzon I Ressler KJ. From candidate genes to genome-wide association: the challenges and promise of posttraumatic stress disorder genetic studies. Biol Psychiatry. (2013) 74:634–6. 10.1016/j.biopsych.2013.08.022 24120289 PMC4617623

[27] Wang S Jiang X Singh S Marmor R Bonomi L Fox D. Genome privacy: challenges, technical approaches to mitigate risk, and ethical considerations in the United States. Ann N Y Acad Sci. (2017) 1387:73–83. 10.1111/nyas.13259 27681358 PMC5266631

[28] Bojarczuk A. Ethical aspects of human genome research in sports-a narrative review. Genes. (2024) 15:1216. 10.3390/genes15091216 39336807 PMC11430849

[29] Caimari A Boqué N Canela N Herrero P Mayneris-perxachs J Arola L. Metabolomics and proteomics as tools to advance the understanding of exercise responses: the emerging role of gut microbiota in athlete health and performance. In: Sports, Exercise, and Nutritional Genomics. Academic Press (2019). p. 433–59. 10.1016/B978-0-12-816193-7.00019-1

[30] Mead MN. Nutrigenomics: the genome–food interface. Environ Health Perspect. (2007) 115:A582–9. 10.1289/ehp.115-a582 18087577 PMC2137135

[31] Farhud D Zarif Yeganeh M Zarif Yeganeh M. Nutrigenomics and nutrigenetics. Iran J Public Health. (2010) 39:1–14. PMC3481686 23113033

[32] Fenech M El-Sohemy A Cahill L Ferguson LR French TAC Tai ES. Nutrigenetics and nutrigenomics: viewpoints on the current status and applications in nutrition research and practice. J Nutrigenet Nutrigenomics. (2011) 4:69–89. 10.1159/000327772 21625170 PMC3121546

[33] Lai CQ Smith CE Parnell LD Lee YC Corella D Hopkins P. Epigenomics and metabolomics reveal the mechanism of the APOA2-saturated fat intake interaction affecting obesity. Am J Clin Nutr. (2018) 108:188–200. 10.1093/ajcn/nqy081 29901700 PMC6454512

[34] Caren ES Katherine LT Donna KA Sabrina EN Dolores C Ingrid BB. Apolipoprotein A2 polymorphism interacts with intakes of dairy foods to influence body weight in 2 U.S Populations. J Nutr. (2013) 143:1865–71. 10.3945/jn.113.179051 24108135 PMC3827635

[35] Ramos-Lopez O Martinez JA Milagro FI. Holistic integration of omics tools for precision nutrition in health and disease. Nutrients. (2022) 14:4074. 10.3390/nu14194074 36235725 PMC9572439

[36] JanssenDuijghuijsen L Looijesteijn E van den Belt M Gerhard B Ziegler M Ariens R. Changes in gut microbiota and lactose intolerance symptoms before and after daily lactose supplementation in individuals with the lactase nonpersistent genotype. Am J Clin Nutr. (2024) 119:702–10. 10.1016/j.ajcnut.2023.12.016 38159728

[37] Ferguson JF Allayee H Gerszten RE Ideraabdullah F Kris-Etherton PM Ordovás JM. Nutrigenomics, the microbiome, and gene-environment interactions: new directions in cardiovascular disease research, prevention, and treatment: a scientific statement from the American Heart Association. Circ Cardiovasc Genet. (2016) 9:291–313. 10.1161/HCG.0000000000000030 27095829 PMC7829062

[38] Guest NS Horne J Vanderhout SM El-Sohemy A. Sport nutrigenomics: personalized nutrition for athletic performance. Front Nutr. (2019) 6:8. 10.3389/fnut.2019.00008 30838211 PMC6389634

[39] Varillas-Delgado D. Influence of genetic polymorphisms and biochemical biomarkers on response to nutritional iron supplementation and performance in a professional football team: a pilot longitudinal study. Nutrients. (2025) 17:1379. 10.3390/nu17081379 40284242 PMC12030593

[40] Muniz-Santos R Magno-França A Jurisica I Cameron LC. From microcosm to macrocosm: the -omics, multiomics, and sportomics approaches in exercise and sports. OMICS. (2023) 27:499–518. 10.1089/omi.2023.0169 37943554

[41] Robbins JM Rao P Deng S Keyes MJ Tahir UA Katz DH. Plasma proteomic changes in response to exercise training are associated with cardiorespiratory fitness adaptations. JCI Insight. (2023) 8:e165867. 10.1172/jci.insight.165867 37036009 PMC10132160

[42] Furrer R Handschin C. Molecular aspects of the exercise response and training adaptation in skeletal muscle. Free Radic Biol Med. (2024) 223:53–68. 10.1016/j.freeradbiomed.2024.07.026 39059515 PMC7617583

[43] Teschler M Mooren FC. (Whole-Body) electromyostimulation, muscle damage, and immune system: a mini review. Front Physiol. (2019) 10:1461. 10.3389/fphys.2019.01461 31849709 PMC6895567

[44] Malsagova KA Kopylov AT Stepanov AA Kulikova LI Izotov AA Yurku KA. Metabolomic and proteomic profiling of athletes performing physical activity under hypoxic conditions. Sports. (2024) 12:72. 10.3390/sports12030072 38535735 PMC10975304

[45] Zallocco L Giusti L Ronci M Mussini A Trerotola M Mazzoni M. Salivary proteome changes in response to acute psychological stress due to an oral exam simulation in university students: effect of an olfactory stimulus. Int J Mol Sci. (2021) 22:4295. 10.3390/ijms22094295 33919012 PMC8122612

[46] Bellagambi FG Lomonaco T Salvo P Vivaldi F Hangouët M Ghimenti S. Saliva sampling: methods and devices. An overview. Trac Trend Anal Chem. (2020) 124:115781. 10.1016/j.trac.2019.115781

[47] Bongiovanni T Lacome M Fanos V Martera G Cione E Cannataro R. Metabolomics in team-sport athletes: current knowledge, challenges, and future perspectives. Proteomes. (2022) 10:27. 10.3390/proteomes10030027 35997439 PMC9396992

[48] Liang D Chen C Huang S Liu S Fu L Niu Y. Alterations of lysine acetylation profile in murine skeletal muscles upon exercise. Front Aging Neurosci. (2022) 14:859313. 10.3389/fnagi.2022.859313 35592697 PMC9110802

[49] Hoffman NJ. Omics and exercise: global approaches for mapping exercise biological networks. Cold Spring Harbor Perspect Med. (2017) 7:a029884. 10.1101/cshperspect.a029884 PMC5629985 28348175

[50] Biçakçi B Cieszczyk P Humińska-Lisowska K. Genetic determinants of endurance: a narrative review on elite athlete status and performance. Int J Mol Sci. (2024) 25:13041. 10.3390/ijms252313041 39684752 PMC11641144

[51] Li W Zhang M Hu Y Shen P Bai Z Huangfu C. Acute mountain sickness prediction: a concerto of multidimensional phenotypic data and machine learning strategies in the framework of predictive, preventive, and personalized medicine. EPMA J. (2025). 10.1007/s13167-025-00404-9 PMC12106293 40438497

[52] Wang G Durussel J Shurlock J Mooses M Fuku N Bruinvels G. Validation of whole-blood transcriptome signature during microdose recombinant human erythropoietin (rHuEpo) administration. BMC Genom. (2017) 18:817. 10.1186/s12864-017-4191-7 PMC5688496 29143667

[53] San-Millán I Stefanoni D Martinez JL Hansen KC D'Alessandro A Nemkov T. Metabolomics of endurance capacity in world tour professional cyclists. Front Physiol. (2020) 11:578. 10.3389/fphys.2020.00578 32581847 PMC7291837

[54] Ou Z Yang L Wu J Xu M Weng X Xu G. Metabolic characteristics of ischaemic preconditioning induced performance improvement in Taekwondo athletes using LC–MS/MS-based plasma metabolomics. Sci Rep. (2024) 14:24609. 10.1038/s41598-024-76045-1 39427043 PMC11490506

[55] Schranner D Kastenmüller G Schönfelder M Römisch-Margl W Wackerhage H. Metabolite concentration changes in humans after a bout of exercise: a systematic review of exercise metabolomics studies. Sports Med Open. (2020) 6:11. 10.1186/s40798-020-0238-4 32040782 PMC7010904

[56] Belhaj MR Lawler NG Hoffman NJ. Metabolomics and lipidomics: expanding the molecular landscape of exercise biology. Metabolites. (2021) 11:151. 10.3390/metabo11030151 33799958 PMC8001908

[57] Evans M Cogan KE Egan B. Metabolism of ketone bodies during exercise and training: physiological basis for exogenous supplementation. J Physiol. (2017) 595:2857–71. 10.1113/JP273185 27861911 PMC5407977

[58] Sakaguchi CA Nieman DC Signini EF Abreu RM Catai AM. Metabolomics-based studies assessing exercise-induced alterations of the human metabolome: a systematic review. Metabolites. (2019) 9:164. 10.3390/metabo9080164 31405020 PMC6724094

[59] Kern F Ludwig N Backes C Maldener E Fehlmann T Suleymanov A. Systematic assessment of blood-borne microRNAs highlights molecular profiles of endurance sport and carbohydrate uptake. Cells. (2019) 8:1045. 10.1101/721928 31500139 PMC6770460

[60] Muscella A Stefàno E Lunetti P Capobianco L Marsigliante S. The regulation of fat metabolism during aerobic exercise. Biomolecules. (2020) 10:1699. 10.3390/biom10121699 33371437 PMC7767423

[61] Ashcroft SP Stocks B Egan B Zierath JR. Exercise induces tissue-specific adaptations to enhance cardiometabolic health. Cell Metab. (2024) 36:278–300. 10.1016/j.cmet.2023.12.008 38183980

[62] Olsen L Thum E Rohner N. Lipid metabolism in adaptation to extreme nutritional challenges. Dev Cell. (2021) 56:1417–29. 10.1016/j.devcel.2021.02.024 33730548

[63] Goodpaster BH Sparks LM. Metabolic flexibility in health and disease. Cell Metab. (2017) 25:1027–36. 10.1016/j.cmet.2017.04.015 28467922 PMC5513193

[64] Qi S Li X Yu J Yin L. Research advances in the application of metabolomics in exercise science. Front Physiol. (2023) 14:1332104. 10.3389/fphys.2023.1332104 38288351 PMC10822880

[65] Yang PL. Metabolomics and lipidomics: yet more ways your health is influenced by fat. In:Katze MG Korth MJ Law GL Nathanson N, editors. Viral Pathogenesis. Academic Press (Elsevier) (2016). p. 181–98. 10.1016/B978-0-12-800964-2.00014-8

[66] Latino F Cataldi S Carvutto R De Candia M D'Elia F Patti A. The importance of lipidomic approach for mapping and exploring the molecular networks underlying physical exercise: a systematic review. Int J Mol Sci. (2021) 22:8734. 10.3390/ijms22168734 34445440 PMC8395903

[67] Ivanisevic T Sewduth RN. Multi-omics integration for the design of novel therapies and the identification of novel biomarkers. Proteomes. (2023) 11:34. 10.3390/proteomes11040034 37873876 PMC10594525

[68] Babu M Snyder M. Multi-omics profiling for health. Mol Cell Proteomics. (2023) 22:100561. 10.1016/j.mcpro.2023.100561 37119971 PMC10220275

[69] Mohr AE Ortega-Santos CP Whisner CM Klein-Seetharaman J Jasbi P. Navigating challenges and opportunities in multi-omics integration for personalized healthcare. Biomedicines. (2024) 12:1496. 10.3390/biomedicines12071496 39062068 PMC11274472

[70] Zheng Y Liu Y Yang J Dong L Zhang R Tian S. Multi-omics data integration using ratio-based quantitative profiling with Quartet reference materials. Nat Biotechnol. (2024) 42:1133–49. 10.1038/s41587-023-01934-1 37679543 PMC11252085

[71] San-Millán I. Blood biomarkers in sports medicine and performance and the future of metabolomics. Methods Mol Biol. (2019) 1978:431–46. 10.1007/978-1-4939-9236-2_26 31119678

[72] Shao Y Lv X Ying S Guo Q. Artificial intelligence-driven precision medicine: multi-omics and spatial multi-omics approaches in Diffuse Large B-Cell Lymphoma (DLBCL). Front Biosci. (2024) 29:404. 10.31083/j.fbl2912404 39735973

[73] Tanaka M. From serendipity to precision: integrating AI, multi-omics, and human-specific models for personalized neuropsychiatric care. (2025). 10.20944/preprints202412.0679.v2 PMC11761901 39857751

[74] Herráiz-Gil S De Arriba M Escámez M Leon C. Multi-omic data integration in food science and analysis. Curr Opin Food Sci. (2023) 52:101049. 10.1016/j.cofs.2023.101049

[75] Usova EI Alieva AS Yakovlev AN Alieva MS Prokhorikhin AA Konradi AO. Integrative analysis of multi-omics and genetic approaches-a new level in atherosclerotic cardiovascular risk prediction. Biomolecules. (2021) 11:1597. 10.3390/biom11111597 34827594 PMC8615817

[76] Shi Z Li X Shuai Y Lu Y Liu Q. The development of wearable technologies and their potential for measuring nutrient intake: towards precision nutrition. Nutr Bullet. (2022) 47:388–406. 10.1111/nbu.12581 36134894

[77] Jonvik KL King M Rollo I Stellingwerff T Pitsiladis Y. New opportunities to advance the field of sports nutrition. Front Sports Act Living. (2022) 4:852230. 10.3389/fspor.2022.852230 35252862 PMC8891369

[78] Sorrenti V Fortinguerra S Caudullo G Buriani A. Deciphering the role of polyphenols in sports performance: from nutritional genomics to the gut microbiota toward phytonutritional epigenomics. Nutrients. (2020) 12:1265. 10.3390/nu12051265 32365576 PMC7281972

[79] Khoramipour K Sandbakk Ø Keshteli AH Gaeini AA Wishart DS Chamari K. Metabolomics in exercise and sports: a systematic review. Sports Med. (2022) 52:547–83. 10.1007/s40279-021-01582-y 34716906

[80] Suleman S Niaz A Akram M Hadi B Usman M Sajjad M. The role of nutrigenomics in sports performance: a quantitative overview of gene-diet interactions. J Health Rehabil Res. (2024) 4:1713–8. 10.61919/jhrr.v4i1.664

[81] Ussher JR Elmariah S Gerszten RE Dyck JRB. The emerging role of metabolomics in the diagnosis and prognosis of cardiovascular disease. J Am Coll Cardiol. (2016) 68:2850–70. 10.1016/j.jacc.2016.09.972 28007146

[82] Matsui T Liu YF Soya M Shima T Soya H. Tyrosine as a mechanistic-based biomarker for brain glycogen decrease and supercompensation with endurance exercise in rats: a metabolomics study of plasma. Front Neurosci. (2019) 13:200. 10.3389/fnins.2019.00200 30941004 PMC6433992

[83] Donnan KJ Williams EL Stanger N. Tyrosine supplementation is ineffective in facilitating soccer players' physical and cognitive performance during high-intensity intermittent exercise in hot conditions. PLoS ONE. (2025) 20:e0317486. 10.1371/journal.pone.0317486 39820592 PMC11737745

[84] Arnold PK Finley LWS. Regulation and function of the mammalian tricarboxylic acid cycle. J Biol Chem. (2023) 299:102838. 10.1016/j.jbc.2022.102838 36581208 PMC9871338

[85] Liu H Wang S Wang J Guo X Song Y Fu K. Energy metabolism in health and diseases. Signal Transduct Target Ther. (2025) 10:69. 10.1038/s41392-025-02141-x 39966374 PMC11836267

[86] Gong X Yang SY Wang ZY Tang M. The role of hypoxic microenvironment in autoimmune diseases. Front Immunol. (2024) 15:1435306. 10.3389/fimmu.2024.1435306 39575238 PMC11578973

[87] Davison G Vinaixa M McGovern R Beltran A Novials A Correig X. Metabolomic response to acute hypoxic exercise and recovery in adult males. Front Physiol. (2018) 9:1682. 10.3389/fphys.2018.01682 30534085 PMC6275205

[88] Patle S Rotake D. Recent advances, technological challenges and requirements to predict the future treads in wearable sweat sensors: a critical review. Microchem J. (2024) 200:110457. 10.1016/j.microc.2024.110457

[89] Assalve G Lunetti P Di Cagno A De Luca EW Aldegheri S Zara V. Advanced wearable devices for monitoring sweat biochemical markers in athletic performance: a comprehensive review. Biosensors. (2024) 14:574. 10.3390/bios14120574 39727839 PMC11674680

[90] Gao F Liu C Zhang L Liu T Wang Z Song Z. Wearable and flexible electrochemical sensors for sweat analysis: a review. Microsyst Nanoeng. (2023) 9:1. 10.1038/s41378-022-00443-6 36597511 PMC9805458

[91] Erdem A Eksin E Senturk H Yildiz E Maral M. Recent developments in wearable biosensors for healthcare and biomedical applications. Trac Trend Analyt Chem. (2024) 171:117510. 10.1016/j.trac.2023.117510

[92] Clark KM Ray TR. Recent advances in skin-interfaced wearable sweat sensors: opportunities for equitable personalized medicine and global health diagnostics. ACS Sensors. (2023) 8:3606–22. 10.1021/acssensors.3c01512 37747817 PMC11211071

[93] Alzahrani A Ullah A. Advanced biomechanical analytics: wearable technologies for precision health monitoring in sports performance. Digit Health. (2024) 10:20552076241256744. 10.1177/20552076241256745 PMC11151756 38840658

[94] Chidambaram S Maheswaran Y Patel K Sounderajah V Hashimoto DA Seastedt KP. Using artificial intelligence-enhanced sensing and wearable technology in sports medicine and performance optimisation. Sensors. (2022) 22:6920. 10.3390/s22186920 36146263 PMC9502817

[95] Wang L Meng Q Su CH. From food supplements to functional foods: emerging perspectives on post-exercise recovery nutrition. Nutrients. (2024) 16:4081. 10.20944/preprints202410.1811.v1 39683475 PMC11643565

[96] Díaz-Lara J Reisman E Botella J Probert B Burke LM Bishop DJ. Delaying post-exercise carbohydrate intake impairs next-day exercise capacity but not muscle glycogen or molecular responses. Acta Physiol. (2024) 240:e14215. 10.1111/apha.14215 39263899

[97] Margolis LM Allen JT Hatch-McChesney A Pasiakos SM. Coingestion of carbohydrate and protein on muscle glycogen synthesis after exercise: a meta-analysis. Med Sci Sports Exerc. (2021) 53:384–93. 10.1249/MSS.0000000000002476 32826640 PMC7803445

[98] Gillen JB West DWD Williamson EP Fung HJW Moore DR. Low-carbohydrate training increases protein requirements of endurance athletes. Med Sci Sports Exerc. (2019) 51:2294–301. 10.1249/MSS.0000000000002036 31083047

[99] Remels AHV Gosker HR Langen RCJ Schols AMWJ. The mechanisms of cachexia underlying muscle dysfunction in COPD. J Appl Physiol. (2012) 114:1253–62. 10.1152/japplphysiol.00790.2012 23019314

[100] Chorell E Moritz T Branth S Antti H Svensson MB. Predictive metabolomics evaluation of nutrition-modulated metabolic stress responses in human blood serum during the early recovery phase of strenuous physical exercise. J Proteome Res. (2009) 8:2966–77. 10.1021/pr900081q 19317510

[101] Glancy B Balaban RS. Energy metabolism design of the striated muscle cell. Physiol Rev. (2021) 101:1561–607. 10.1152/physrev.00040.2020 33733879 PMC8576364

[102] Ru Q Li Y Zhang X Chen L Wu Y Min J. Iron homeostasis and ferroptosis in muscle diseases and disorders: mechanisms and therapeutic prospects. Bone Res. (2025) 13:27. 10.1038/s41413-024-00398-6 40000618 PMC11861620

[103] Nieman DC Groen AJ Pugachev A Simonson AJ Polley K James K. Proteomics-based detection of immune dysfunction in an elite adventure athlete trekking across the Antarctica. Proteomes. (2020) 8:4. 10.3390/proteomes8010004 32138228 PMC7151708

[104] Li X Yang Y Zhang B Lin X Fu X An Y. Lactate metabolism in human health and disease. Signal Transduct Target Ther. (2022) 7:305. 10.1038/s41392-022-01151-3 36050306 PMC9434547

[105] Torma F Gombos Z Jokai M Takeda M Mimura T Radak Z. High intensity interval training and molecular adaptive response of skeletal muscle. Sports Med Health Sci. (2019) 1:24–32. 10.1016/j.smhs.2019.08.003 35782463 PMC9219277

[106] Scott BR Goods PSR Slattery KM. High-intensity exercise in hypoxia: is increased reliance on anaerobic metabolism important? Front Physiol. (2016) 7:637. 10.3389/fphys.2016.00637 28082907 PMC5186758

[107] Bennett S Brocherie F Phelan MM Tiollier E Guibert E Morales-Artacho AJ. Acute heat stress amplifies exercise-induced metabolomic perturbations and reveals variation in circulating amino acids in endurance-trained males. Exp Physiol. (2023) 108:838–51. 10.1113/EP090911 36691850 PMC10988456

[108] Fuchs CJ Gonzalez JT van Loon LJC. Fructose co-ingestion to increase carbohydrate availability in athletes. J Physiol. (2019) 597:3549–60. 10.1113/JP277116 31166604 PMC6852172

[109] Jeukendrup A. A step towards personalized sports nutrition: carbohydrate intake during exercise. Sports Med. (2014) 44(Suppl 1):S25–33. 10.1007/s40279-014-0148-z 24791914 PMC4008807

[110] Li Q Wang J Zhao C. From genomics to metabolomics: molecular insights into osteoporosis for enhanced diagnostic and therapeutic approaches. Biomedicines. (2024) 12:2389. 10.3390/biomedicines12102389 39457701 PMC11505085

[111] Grabia M Perkowski J Socha K Markiewicz-Żukowska R. Female athlete triad and relative energy deficiency in sport (REDs): nutritional management. Nutrients. (2024) 16:359. 10.3390/nu16030359 38337644 PMC10857508

[112] Helm MM McGinnis GR Basu A. Impact of nutrition-based interventions on athletic performance during menstrual cycle phases: a review. Int J Environ Res Public Health. (2021) 18:6294. 10.3390/ijerph18126294 34200767 PMC8296102

[113] Manore MM. Dietary recommendations and athletic menstrual dysfunction. Sports Med. (2002) 32:887–901. 10.2165/00007256-200232140-00002 12427050

[114] Holtzman B Ackerman KE. Recommendations and nutritional considerations for female athletes: health and performance. Sports Med. (2021) 51:43–57. 10.1007/s40279-021-01508-8 34515972 PMC8566643

[115] Bailey RL Dog TL Smith-Ryan AE Das SK Baker FC Madak-Erdogan Z. Sex differences across the life course: a focus on unique nutritional and health considerations among women. J Nutr. (2022) 152:1597–610. 10.1093/jn/nxac059 35294009 PMC9258555

[116] Ceylan H. Nutritional strategies for peak performance: guidelines for athletes' optimal fueling and recovery. Health Nexus. (2023) 1:90–8. 10.61838/kman.hn.1.4.11

[117] Guo Y Wang S Chao X Li D Wang Y Guo Q. Multi-omics studies reveal ameliorating effects of physical exercise on neurodegenerative diseases. Front Aging Neurosci. (2022) 14:1026688. 10.3389/fnagi.2022.1026688 36389059 PMC9659972

[118] Jaguri A Al Thani AA Elrayess MA. Exercise metabolome: insights for health and performance. Metabolites. (2023) 13:694. 10.3390/metabo13060694 37367852 PMC10305288

[119] Glass KA Germain A Huang YV Hanson MR. Urine metabolomics exposes anomalous recovery after maximal exertion in female ME/CFS patients. Int J Mol Sci. (2023) 24:3685. 10.3390/ijms24043685 36835097 PMC9958671

[120] Appel M Zentgraf K Krüger K Alack K. Effects of genetic variation on endurance performance, muscle strength, and injury susceptibility in sports: a systematic review. Front Physiol. (2021) 12:694411. 10.3389/fphys.2021.694411 34366884 PMC8334364

[121] Varillas-Delgado D Del Coso J Gutiérrez-Hellín J Aguilar-Navarro M Muñoz A Maestro A. Genetics and sports performance: the present and future in the identification of talent for sports based on DNA testing. Eur J Appl Physiol. (2022) 122:1811–30. 10.1007/s00421-022-04945-z 35428907 PMC9012664

[122] Shang Z Pai L Patil S. Unveiling the dynamics of gut microbial interactions: a review of dietary impact and precision nutrition in gastrointestinal health. Front Nutr. (2024) 11:1395664. 10.3389/fnut.2024.1395664 38873568 PMC11169903

[123] Hayes CN Nakahara H Ono A Tsuge M Oka S. From omics to multi-omics: a review of advantages and tradeoffs. Genes. (2024) 15:1551. 10.20944/preprints202411.0882.v1 39766818 PMC11675490

